# Social context affects tail displays by *Phrynocephalus vlangalii* lizards from China

**DOI:** 10.1038/srep31573

**Published:** 2016-08-16

**Authors:** Richard A. Peters, Jose A. Ramos, Juan Hernandez, Yayong Wu, Yin Qi

**Affiliations:** 1 Animal Behaviour Group Department of Ecology, Environment & Evolution La Trobe University, Melbourne, Victoria, Australia; 2 Department of Herpetology Chengdu Institute of Biology, Chengdu, Sichuan, China

## Abstract

Competition between animals for limited resources often involves signaling to establish ownership or dominance. In some species, the defended resource relates to suitable thermal conditions and refuge from predators. This is particularly true of burrow-dwelling lizards such as the Qinghai toad-headed agama (*Phrynocephalus vlangalii*), which are found on the Tibetan plateau of western China. Male and female lizards occupy separate burrows, which are vital for anti-predator behaviour during warmer months when lizards are active and, crucially, provide shelter from harsh winter conditions. These lizards are readily observed signaling by means of tail displays on the sand dunes they inhabit. Given the selective pressure to hold such a resource, both males and females should exhibit territorial behaviour and we considered this study system to examine in detail how social context influences motion based territorial signaling. We confirmed that territorial signaling was used by both sexes, and by adopting a novel strategy that permitted 3D reconstruction of tail displays, we identified significant variation due to social context. However, signal structure was not related to lizard morphology. Clearly, the burrow is a highly valued resource and we suggest that additional variation in signaling behaviour might be mediated by resource quality.

Animals compete when resources are limited, and such contests have been considered widely in the context of foraging and mating^1^. When this competition relates to a specific area in space, where food is abundant, for example, we consider resource holders to be exhibiting territoriality^2^. Territorial behaviour need not always involve physical contact that carries the risk of injury^3^ or even death^4^. Indeed many species produce signals to resolve conflict without the need for physical contact^5^, and the structure of these signals is predicted to contain sufficient information from which receivers can decide whether escalating the contest is worthwhile^6^,^7^.

Lizards are well known for their territorial signals, which vary from conspicuous coloration and movements^8^,^9^, to chemical cues^10^ and even acoustic signals^11^. The territories of many species are centred on the distribution of food resources, such that territory size is positively correlated with the availability of food^12^. However, in some lizard species, food availability seems to play a lesser role^13^, with thermal conditions and refuge from predators providing the key resources those animals seek to defend^14^. This is particularly true of burrow dwelling species, whereby the burrow entrance is the centre of their territory and vigorously defended from potential usurpers. One such species is the Qinghai toad-headed agama (*Phrynocephalus vlangalii;* Fig. 1a), which is a high-elevation viviparous lizard found on the Tibetan Plateau in northwest China. These lizards live in high densities and occupy small overlapping home ranges centred on a burrow^15^. Burrows are a vital resource for these lizards, providing a refuge from predators during spring and summer, and a shelter from harsh winter conditions when the ground is covered in thick snow. Males aggressively defend their burrows. Indeed it is reported that variance in reproductive success is linked to the control of space with males in better condition occupying burrows that overlap with more female burrows^15^ that presumably provides greater access to females and opportunities for mating.

Visual displays between residents and intruders are a conspicuous feature of male *P. vlangalii* behaviour at their burrow. These displays involve movement of the tail and are more frequently used by burrow owners than floaters^16^, suggesting they likely function to establish territory ownership. Furthermore, as dynamic displays are expected to be costly^17^ they also might provide information about signaler quality. Although there is little evidence to suggest that the frequency of male signaling bouts by *P. vlangalii* is condition dependent^16^, we postulated that variation in signal structure, rather than signal frequency, might hold key information for receivers regarding individual quality. Attempts to relate signals with sender quality is not straightforward^18^, but one possible explanation suggests that non-significant relationships arise because of the difficulty in measuring signals^19^. Consequently, attempts to relate tail displays with sender characteristics will require careful, detailed analyses of signal structure.

The strong selective pressure on males of shelter-based species to defend their key resource^20^ must also be true for females. Indeed female resource defense behaviour is reported in a variety of taxonomic groups in which the ownership, control and defense of a key resource have clear functional benefits for females. Examples of territorial females include the funnel web building spider, *Agelenopsis aperta*, in defense of her web^21^,^22^, the fidder crab, *Uca vocans,* that vigorously defends her burrow and the space surrounding it^23^, and the lizard, *Iguana iguana* that fights off other females from her chosen nesting site^24^. The behaviour of these species suggests a valued resource, but the level of territorial behaviour exhibited will vary according to the perceived value of the resource. For example, female fiddler crabs, *U. pugilator*, utilize burrows for reproduction but do not aggressively defend them (Christy 1980, cited by Salmon 1984). The incubation burrows that females require are functionally important but are controlled by males and abundant on the mudflats they inhabit^25^. The cost of defense in this case clearly outweighs any benefit. In the cases where female territoriality is reported, communicative displays do form part of the defense. Female *U. vocans* lack the enlarged claw used by males in signaling but still perform waving displays to rivals, while *A. aperta* generate visual displays when intruders are at close range^21^. In both cases, however, displays are uncommon. Female fiddler crabs have limited capacity to generate conspicuous signals, while funnel-web building spiders utilize vibration cues transmitted through the web as the intruder moves around that reveals body size information that is an important predictor of contest outcome^21^. Displays seem more important to nest defense by *I. iguana*, with females having the option of simple and elaborate displays, but ultimately, it is the willingness to invest in high energy displays that predicts outcome^24^. In this way, female displays can provide information about the relative value of the resource and motivation of the respective participants. Female *P. vlangalii* chase away conspecifics in the vicinity of their burrow entrances, but are also reliably observed performing tail displays near to their burrow entrance. However, it is not known whether these signals function in resource defense; if so, how do they vary from that of males? Unlike *I. iguana* females that defend resources from other females, *P. vlangalii* exist in mixed sex colonies and are likely to need to defend their resource from both sexes. Frequent interactions between different sexes and age classes, combined with the high value placed on burrows, points toward a complex signaling system^26^. Consequently, *P. vlangalii* provides a nice system in which to examine how social context affects signaling behaviour, with a specific focus on the use and structure of movement-based signals.

Therefore, our goal in the present study was to examine burrow defense by *P. vlangalii,* and to confirm the use of signals as part of this defense. Furthermore, we wished to examine whether social context affects signaling and if signal structure is related to qualities of the signaler. Given the functional importance of burrows to all lizards, we hypothesized that females would also signal in defense of their resource and duly compared and contrasted the signaling behaviour of male and female lizards. We used tethered intruders from separate study sites and filmed responses by residents as a function of the sex and age class of both residents and the intruders. We first identified the different types of tail displays and how these were used in different contexts. By filming displays using two cameras we reconstructed movements in three-dimensions (3D) and calculated amplitudes and speeds from these reconstructions to quantify signal structure in greater detail than in previous studies. This was important as it ensured we were not limited to a single camera view, which can have important consequences for the perceived signal^27^. Morphological measurements of lizards were undertaken and examined in relation to our measurements of signal structure. By presenting unfamiliar intruders we assumed interactions reflected first meetings between resident-intruder dyads and thereby avoids the possibility of social recognition mediating behaviour, which incorporates individual recognition and past experience^19^ and might not have encouraged full displays. We predicted that signal use would vary between the sexes, and between adults and juveniles, and that tail movements would reflect signaler phenotype.

## Results

### Signal use by resident and intruder type

Tail displays were generated in 91 out of the 108 trials and three different types were recorded (Fig. 2). Almost half of the trials in which signaling was not observed (8/17) involved adult male or female residents paired with a juvenile intruder (Fig. 3b,c). In these trials, adult lizards approached the juvenile intruder, which quickly retreated and the trial was terminated. When signaling did occur, there was a clear pattern in the use of the different tail displays. Juvenile lizards predominantly exhibited tail waving, although tail coiling was observed on 3/36 trials (Fig. 3a). Tail waving by females was observed twice throughout the study (Fig. 3b), while both female (Fig. 3b) and male lizards (Fig. 3c) generated tail coiling displays, and only males exhibited tail lashing (Fig. 3c). However, tail lashing was used only in just over half of the trials involving male residents (19/36). The probability of generating this type of display was not predicted by resident or intruder weight and SVL, as the 95% confidence intervals for each fixed effect spanned zero (Table 1).

### Variation in tail coiling by adult male and female residents in response to different intruder types

**Figure 1:**
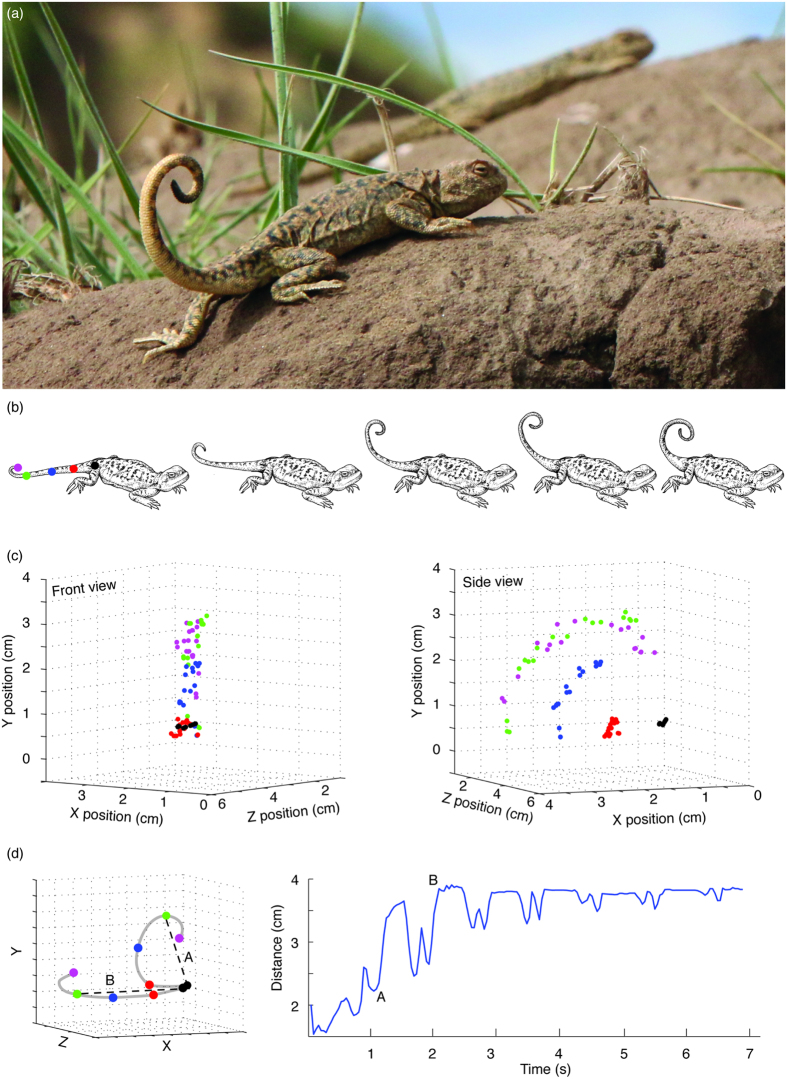
(**a**) Male *Phrynocephalus vlangalii* performing a tail coiling display on a raised mound near to his burrow. Males can be distinguished from females by the black tip of the tail, which is only seen on males. A female *P. vlangalii* is visible in the background. (**b**) Schematic illustration of a tail coiling sequence. The first drawing shows the location of tracking points used in display analysis. (**c**) The position of each point is tracked in every frame from two camera views and a 3D reconstruction of movement is achieved using calibration coefficients (see text for details). (**d**) *Left panel:* Coil amplitude was quantified by measuring the distance between the fourth tracked point (shown in green) with the base of the tail (black). *Right panel:* Plot of coil amplitude by time in which the tail starts out tightly coiled with short distances between point 4 and the tail base (A) and gradually raising and lowering the tail until it is held almost straight (B).

Variation in tail coiling by resident and intruder type is shown in Fig. 4. The results of linear mixed effect models indicated that signal duration (Fig. 4a) and the number of tail raises (Fig. 4b) did not vary as a function of resident or intruder type (Table 2a). The average speed of tail coiling (Fig. 4b) exhibited greater variation, with a significant main effect for resident type indicating that male lizards signal faster than females regardless of intruder type. Coil amplitudes by resident and intruder type are shown in Fig. 4d, and represent the minimum height of point 4 from the base of the tail, such that smaller amplitudes reflect a more coiled tail (Fig. 1b–d). A linear mixed effects model revealed that amplitudes varied by intruder type (Table 2a), with pairwise comparisons showing that amplitudes were significantly greater when the intruder was a juvenile lizard compared to both male and female lizards (Table 2a). This means that adult lizards generate more pronounced coils when the intruder is another adult.

### Variation in tail lashing by adult male residents in response to different intruder types

The average speed of movement during tail lashing by male lizards for the five points along the tail is shown in Fig. 5 separately for each intruder type. Linear mixed effects models revealed a significant main effect for points (Table 2b), although this is not surprising given the kinematics of tail movement and so we did not explore this any further. However, tail speed also varied significantly as a function of intruder type (Table 2b), with pairwise contrasts indicating the males signaled faster when the intruder was another male compared with a female intruder (Table 2b). We considered the relationship between male morphology and tail speed, selecting point 4 along the tail (see Fig. 1b) and on the basis of results above (Fig. 5). Neither SVL or weight was related to signal speed (F_1,7_ = 0.002, p = 0.964 and F_1,7_ = 1.021, p = 0.346 respectively), while tail length suggested a trend toward longer tails producing faster speeds but the result was not significant (F_1,7_ = 5.03, p = 0.060).

## Discussion

Our study confirmed that tail displays are used by *Phrynocephalus vlangalii* lizards in defense of burrows and, importantly, that they are performed by females equally as often as males. However, signal use varied between males and females, as well as between adults and juvenile lizards. A high proportion of juvenile lizards and a few female lizards performed tail-waving displays. We suspect that the tail waving displays performed by juvenile lizards represents a submissive signal to appease intruders. The substantial size difference between adults and juveniles more than explains their submissive behaviour in these contexts, while avoiding conflict with age-matched conspecifics is expected given juveniles share burrows with other juvenile lizards. Adults did not always signal in response to a juvenile intruder, but the majority of interactions between adults did reliably result in tail coiling by both sexes, while about half of the males also performed tail lashing displays. Our approach allowed us to reconstruct signals in 3D and to consider movements in greater detail. Relative to interactions with juveniles, adult male and female lizards generated more pronounced coils that were lowered closer to the body when faced with an adult intruder. The duration of tail coiling, and the number of times lizards raised and lowered their coiled tails did not vary between male and female residents, or as a function of intruder type. However, tail coiling by males was significantly faster than females irrespective of intruder type. Tail lashing was only performed by males and signaling speed varied as a function of intruder type, with males signaling faster in response to other males.

We interpreted tail coiling and tail lashing by *P. vlangalii* to be aggressive displays used in burrow defense, and anticipated that the structure of these signals would vary between individuals in a manner that might reflect motivation or resource holding potential. However, we did not identify significant relationships between lizard morphology and signal characteristics. For tail coiling, we found no relationship between individual characteristics and the resultant signal for either males or females. Similarly, tail lashing by males did not exhibit strong relationships with signaler characteristics, with only a trend for longer tails leading to faster tail movements. This marginally non-significant result is consistent with variation in signaling speeds across *Phrynocephalus* species more generally (Qi, Whiting, Noble, Peters unpublished data), and is largely predicted by the kinematics of the lashing tail. Therefore, signaler quality does not seem to be reflected in the structure of the initial bout (this study), or in the frequency of signaling bouts^16^. Although this general result has been reported in studies of a variety of species, from fish^28^ to birds^29^,^30^, one explanation for not observing condition-dependent signaling is that we did not examine signaling in the right context^31^. It is entirely possible that signals of quality are more important when lizards are establishing territories rather than maintaining territories where groups are relatively stable and interactions are usually mediated by social recognition^19^. Characteristics of the signaler also did not predict the use of tail lashing by males and as such the circumstances that do and do not lead to tail lashing by males require further consideration. The vigorousness of the signal relative to other movements, and the finding that males signal faster in the presence of another male, are both consistent with the hypothesis that tail lashing represents elevated aggression on the part of the resident and that its use is contingent on characteristics of the defended resource. We did not map burrows in our study site and therefore do not know whether some male burrows might be more valuable than others in terms of their position in the landscape, the number of surrounding burrows occupied by females, or the proximity to rival males. Similarly, the internal structure of the burrow might be relevant in predicting the use of a more aggressive signal and warrants further investigation^25^.

**Figure 2:**
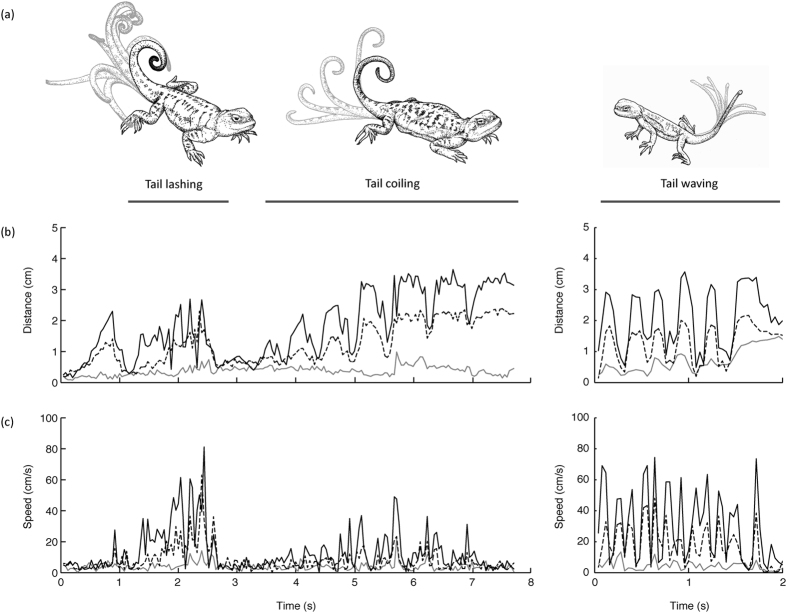
Three different types of tail displays were performed by *P. vlangalii*: tail lashing, tail coiling and tail waving. (**a**) Illustration of the sequence of movements that characterizes each of the different displays depicting a male tail lashing, a female toil coiling and a juvenile generating a tail waving display. (**b**) Display action pattern (DAP) graphs showing the change in position of the middle three tracked points (grey line: point 2; dashed line: point 3; solid line: point 4) relative to the respective starting positions of each point. *Left panel:* Tail lashing followed by tail coiling by a male *P. vlangalii*. In this sequence, the lizard starts with its tail coiled and elevated. It lowers it slightly at around 1 s before commencing tail lashing and returning to a raised and coiled position around 3 s. The lizard then performs tail coiling from the raised position repeatedly raising and lowering the tail before it finishes with an almost fully extended tail. *Right panel:* DAP of a juvenile tail waving display. (**c**) Speed-time graphs of points 2–4 for the sequence shown in (**b**). This example illustrates that tail lashing is characterized by faster speeds than tail coiling (*left panel*), and tail waving by juveniles also involves fast movements (*right panel*).

An interesting feature of our results was the behaviour of adult females. Studies of female display behaviour in lizards have received comparatively less attention, but when they have been the focus of investigation, the data usually shows that females display less frequently^32^,^33^,^34^. In contrast to previous studies of lizards, female *P. vlangalii* were just as likely to perform displays as males and this included same sex interactions. In explaining reduced territorial displays by female *Anolis carolinensis* when interacting with other females, Jenssen *et al*. concluded that there is low inter-female territoriality in this species^32^. The willingness of female *P. vlangalii* to display in response to female intruders in our study leads to the opposite conclusion; that resource defense is just as important for females as it is for males. Previous studies of signaling by female lizards also suggest sex differences in the types of signals used and the intensity of signals^34^,^35^. Orrell and Jenssen showed the *A. carolinensis* females predominantly used two of three display types during interactions, while males predominantly used the third display type^35^. In *P. vlangalii* male and female lizards both use tail coiling signals but males appear to have the option of an additional/alternative component, tail lashing, for reasons that are not yet clear (as described above). However, consistent with previous work is the finding that males display more vigorously than females. Tail lashing is a more vigorous movement (Fig. 2), but even when comparing tail coiling, males signal at higher speeds than females. It is important to note that our study differs a little from previous studies in that we focused specifically on the initial response to an unfamiliar intruder rather than quantifying display behaviour over a longer period of time. As a consequence, female territorial behaviour is currently being investigated in detail, but preliminary data and our own observations during the present study do suggest that female territorial behaviour in *P. vlangalii* is a feature of this system.

Our goal was to examine the use of tail displays in the defense of burrows by *P. vlangalii*. Our findings highlight the importance of burrows to individuals of this species and that territorial displays are a key feature of their defense by both males and females. Like previous studies^29^,^30^,^35^, we have not been able to determine a link between display structure and signaler characteristics, and suggest that signaling behaviour might be more closely linked with resource quality. *Phrynocephalus vlangalii* and other burrow dwelling species lend themselves to such studies as the resource being defended is seemingly easier to characterize and amenable to experimental manipulation, such as altering its proximity to female burrows. Our study also utilizes a more detailed approach to quantifying signal structure that avoids limitations inherent to previous work and encourage others to invest the time to more accurately quantify motion displays.

## Methods

### Study site and study animals

We studied *P. vlangalii* at the Xiaman Conservation Station in the Zoige Wetland Nature Reserve in northwestern China during June of 2014. Three sites featuring sparsely vegetated sand dunes were identified for this study (site A: 33°42′50″N, 102°29′11″E; site B: 33°42′54″N, 102°29′21″E; site C 33°42′44″N, 102°29′18″E), which were between 250 and 310 m apart and separated by contiguous grasses. The grasslands separating these sites do not form a physical barrier between sites, but movement between sites by adults was not observed during the course of the study. Site fidelity and migration between sites is the subject of separate long-term study^15^.

### Experimental design and procedure

Our objective was to observe and quantify signaling behaviour of male, female and juvenile *P. vlangalii* lizards at their burrows in response to male, female and juvenile intruders in a repeated measures design. We captured four males, four females and four juveniles at each of our three sites providing a sample of 36 resident lizards. Additional lizards (14 males, 14 females and 14 juveniles) were captured from the three sites to serve as intruders. Intruders were used three times or less and were not used as residents at any stage during the experiment. We weighed each lizard and measured snout-vent length and tail length to the nearest mm with rulers immediately after capture. To facilitate the subsequent analysis of tail movements from video footage, we marked tails of residents with non-toxic markers at the base of the tail and at three other points along the tail. The distance between points was determined independently for each lizard based on tail length. These three points, along with the base and tip of the tail (which was not marked) provided 5 points to track (Fig. 1b). Lizards were then released and the location of their burrows was marked using a chopstick placed beside the burrow.

We returned to the burrows at least 24 h later to introduce tethered intruders. Male, female and juvenile intruders were presented in a random order, with presentation order counterbalanced across the entire sample. To minimize the effect of individual recognition, intruders for a given resident were selected from one of the other two sites. The responses of residents were filmed with two cameras (Canon Legria HF21 camcorders), and at the conclusion of each trial, a calibration object was placed in the scene and in view of both cameras before recording stopped (Fig. S1). Trials were terminated after the resident completed the first bout of signalling. We waited at least 15 min before presenting the next intruder, based on natural display rates of one every 20 min^16^. Our design resulted in a total of 108 trials between residents and intruders (three trials for each of 36 residents).

### Signal analysis

**Figure 3:**
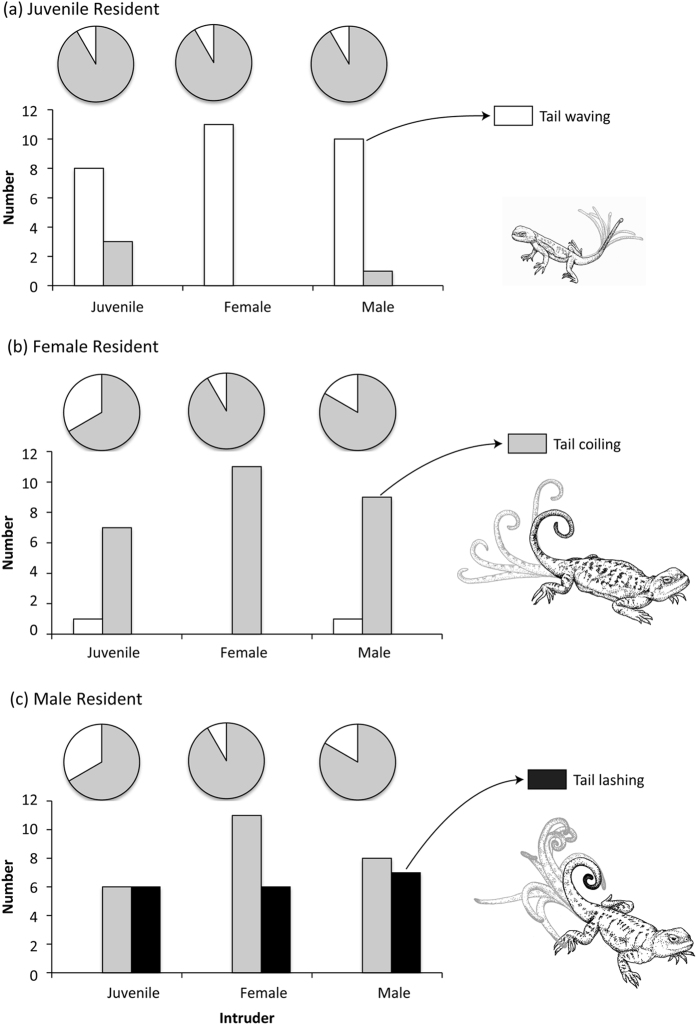
Tail signaling was observed by resident (**a**) juvenile, (**b**) female and (**c**) male *Phrynocephalus vlangalii* at their burrows. Pie charts show the proportion of pairings that resulted in a tail display (shaded). Three different types of tail displays were observed and the relative number of lizards generating each type of display during the initial bout by resident and intruder identity is shown in bar charts. Juveniles primarily generated tail waving (white bars), females generated tail coiling displays (gray), while males generated tail coiling (gray) and tail lashing (black) displays.

**Figure 4:**
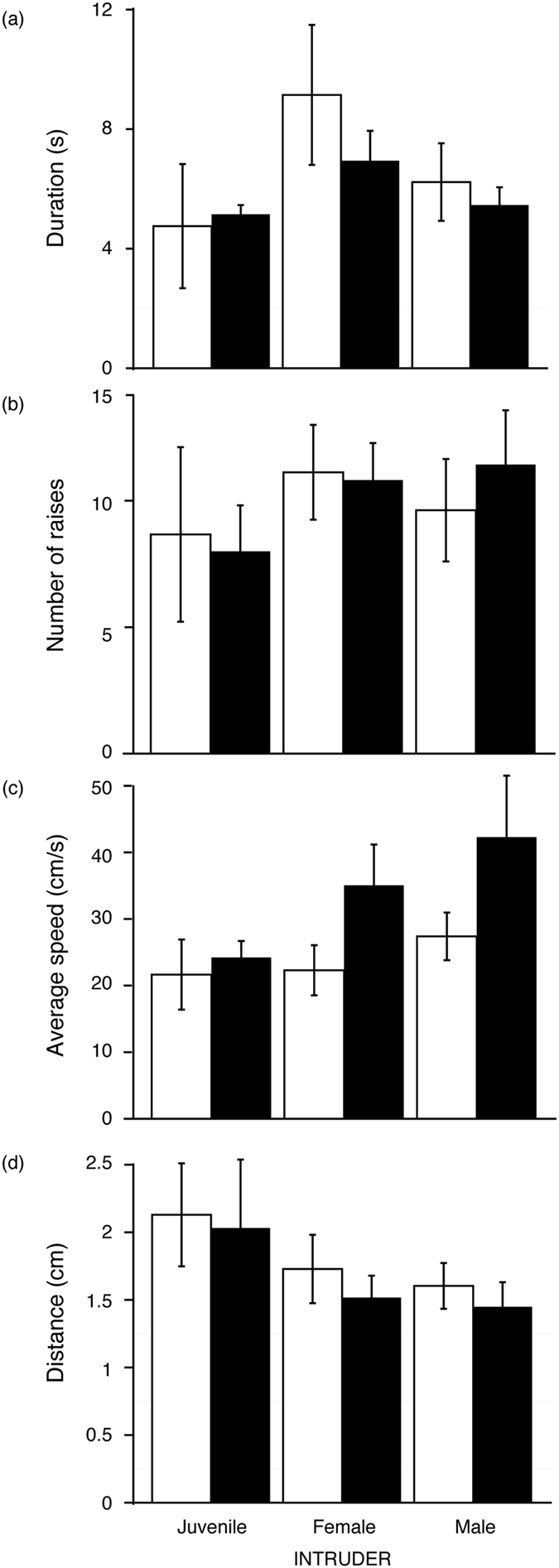
Summary of results comparing tail coiling displays by adult female (white bars) and male (black bars) resident *P. vlangalii* lizards. Responses by residents when exposed to juvenile, female and male intruders were considered in terms of the (**a**) duration of tail coiling, (**b**) number of tail raises, (**c**) average speed of movement and (**d**) coil amplitude, which quantified the minimum distance between the coiled part of the tail and the base of the tail such that shorter distances reflect more pronounced coils. Values shown are means and standard errors.

**Figure 5:**
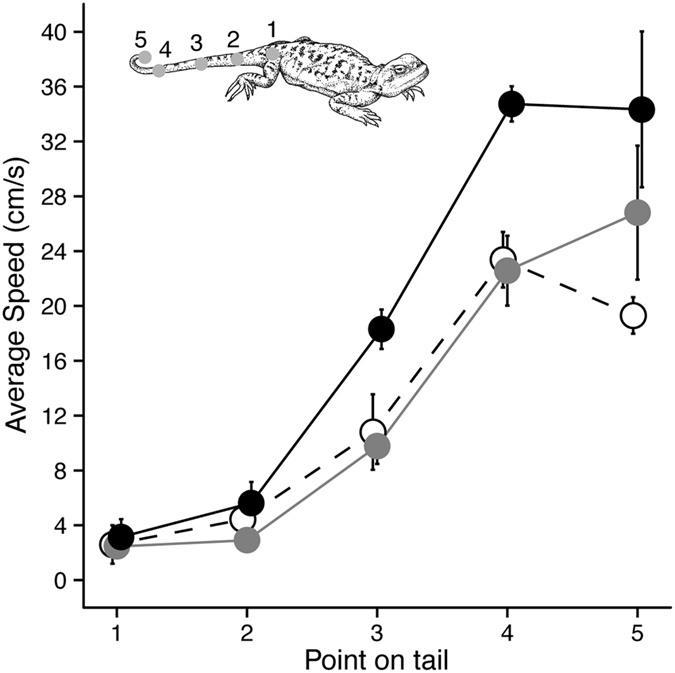
The average speed of movement for each point along the tail during tail lashing by male *P. vlangalii* in response to juvenile (open circles, dashed line), female (gray) and male (black) intruders. Values represent means and standard errors.

Our focus for the present study was the initial response to an unfamiliar intruder rather than to examine the whole interaction. We identified three different tail displays utilised by resident *P. vlangalii* lizards during social interactions at their burrows, which we denote as waving, coiling and lashing (Fig. 2). The use of each of these motor patterns by resident lizards was determined for each trial. We then extracted video footage of these tail displays and reconstructed the movements in 3D using footage from both cameras. Video cameras were calibrated in Matlab (MathWorks Inc.) using direct linear transformation (DLT; following Hedrick^36^) using our calibration object that featured 20 points distributed at different depths and heights throughout the volume of the object (see Supplementary Fig. S1). Clearly identifiable points on the object were located in images from both cameras and digitized to define calibration coefficients (see Hedrick 2008). Footage from each camera was read into Matlab and the position of each of the five points along the tail was located in each frame. The x-y coordinate data for these points was then combined with the DLT calibration coefficients to reconstruct the movement of each point in 3D (Fig. 3c). Some tail displays were excluded from 3D reconstruction, as technical issues during filming would not permit reliable reconstruction of signal structure.

We analyzed tail coiling and tail lashing in more detail in several ways. For tail coiling, we calculated the duration of movement and counted the number of times the tail was raised above the lizard’s body (see Fig. 1b). Another measure of potential variability in tail coiling was assessed by measuring the distance between the fourth point and the base of the tail (henceforth referred to as coil amplitude). As illustrated in Fig. 1d, shorter distances reflect tails that are more tightly coiled and positioned just above the base of the tail; longer distances reflect tails that are straighter and further away from the body. We also calculated the average speed of movement for both tail coiling and tail lashing. For each of our five points, we computed the change in position in 3D space (Euclidean distance) between successive frames (Fig. 1c), and then computed the average speed of movement in cm per frame, which we converted to cm/s by multiplying by 25 (PAL frame rate).

### Statistical analysis

#### Signal use by resident and intruder type

We summarize variation using pie charts to depict the proportion of trials that resulted in a tail display, and frequency histograms to indicate how the different tail displays are used. We examined statistically the probability of tail lashing by males using the *glmer* function in the *lme4* package^37^ in the R statistical Environment^38^. Resident and intruder weight and SVL were used as fixed effects, lizard identity fitted as a random effect (intercept only) along with a binomial error distribution. We examined estimates of the fixed effects from the fitted model with 95% confidence intervals to determine the relative importance of each factor.

#### Variation in tail coiling by adult male and female residents in response to different intruder types

The initial tail coiling displays of 11 males and eight females in the three contexts were examined in detail. We used the same statistical approach for comparing tail coil duration, the number of tail raises, coil amplitude, and average movement speed. In each case, we used the *lmer* function in the *lme4* package in the R statistical Environment. As fixed effects we used resident type (male, female) and intruder type (male, female, juvenile), while lizard identity was used as the random effect (intercept only). The significance of fixed effects, and the interaction between fixed effects, was obtained from the model (F-ratio) and when significant we examined pairwise contrasts from the model.

#### Variation in tail lashing by adult male residents in response to different intruder types

The average speed of movement during the initial tail lashing display by six males across the three contexts was also examined using the *lmer* function in the *lme4* package in the R statistical Environment. We used fixed effects of intruder type (male, female, juvenile) and point on the tail (1–5), and lizard identity as the random effect (intercept only). The significance of fixed effects, and the interaction between fixed effects, was obtained from the model (F-ratio) and when significant we examined pairwise contrasts from the model. The relationship between tail speed and aspects of male morphology (SVL, weight and tail length) were considered using the same function described above, with each variable considered separately as a fixed effect and lizard identity fitted as a random effect in each case. We selected point 4 along the tail (Fig. 1b) and used the speed of movement at this point as our dependent variable.

### Ethical note

The Forestry Department of the Sichuan Provincial Government and the Management Office of the Zoige Nature Reserve approved all fieldwork. Handling of lizards followed approved protocols from the Chengdu Institute of Biology of the Chinese Academy of Sciences, and our activities adhered to the ABS/ASAB “Guidelines for the treatment of animals in Behavioural research and teaching”.

## Additional Information

**How to cite this article**: Peters, R. A. *et al*. Social context affects tail displays by *Phrynocephalus vlangalii* lizards from China. *Sci. Rep.* **6**, 31573; doi: 10.1038/srep31573 (2016).

## Supplementary Material

Supplementary Information

Supplementary Movie S1

## Figures and Tables

**Table 1:** Regression coefficients and associated confidence intervals for resident and intruder weight and snout-vent length (SVL) as predictors of the occurrence of tail lashing by male *P*. *vlangalii.*

	Estimate	95% Confidence Interval	
		Lower	Upper
Intercept	14.84	−40.27	69.94
Resident weight	0.01	−2.54	2.57
Resident SVL	−0.06	−1.23	1.11
Intruder weight	2.05	−0.15	4.25
Intruder SVL	−0.44	−0.92	0.05

**Table 2:** Summary of linear mixed effects models examining tail coiling in adult male and female *P. vlangalii* and tail lashing by adult male *P. vlangalii.*

	Tests of Main Effects				Paired contrasts		
	DF_Num_^a^	DF_Den_^a^	F statistic	P-value	T	DF	P-value
**(a) Tail coiling by male and female lizards**							
Duration							
Resident^b^ × Intruder^c^	2	17	1.194	0.327			
Resident	1	22	0.717	0.406			
Intruder	2	17	2.378	0.123			
Number of tail raises							
Resident × Intruder	2	17	0.338	0.718			
Resident	1	22	0.492	0.490			
Intruder	2	17	1.220	0.320			
Average speed							
Resident × Intruder	2	17	0.504	0.613			
**Resident**	**1**	**22**	**4.806**	**0.039**			
Intruder	2	17	2.156	0.146			
Coil amplitude							
Resident × Intruder	2	16	1.003	0.389			
Resident	1	22	0.636	0.434			
**Intruder**	**2**	**16**	**4.485**	**0.028**			
**Male v Juvenile**					**2.985**	**16**	**0.009**
**Female v Juvenile**					**2.626**	**16**	**0.018**
Male v Female					0.414	16	0.684
**(b) Tail lashing by male lizards**							
Average speed							
Point of tail × Intruder	4	31	0.770	0.552			
**Point of tail**	**2**	**31**	**23.34**	<**0.001**			
**Intruder**	**2**	**31**	**9.400**	**0.0006**			
Male v Juvenile					1.417	31	0.166
Female v juvenile					0.958	31	0.345
**Male v Female**					**5.688**	**31**	**0.012**

Bold values represent significant effects.

^a^DF_Num_ = Numerator degrees of freedom; DF_Den_ = Denominator degrees of freedom.

^b^Male and female.

^c^Male, female and Juvenile.
