# Supplementary material for: Social context affects tail displays by Phrynocephalus vlangalii lizards from China
Source: Sci Rep. 2016 Aug 16;6:31573. doi: 10.1038/srep31573 (PMC4985696; doi:10.1038/srep31573)
Supplement: Supplementary Information [file srep31573-s1.pdf]

Supplementary Information (Figure S1, Movie S1)

Social context affects tail displays by *Phrynocephalus vlangalii* lizards from China

Richard A Peters [1], Jose A Ramos [1], Juan Hernandez [1],

Yayong Wu [2] and Yin Qi [2,*]

[1] Animal Behaviour Group

Department of Ecology, Environment & Evolution

La Trobe University, Melbourne

Victoria, AUSTRALIA

[2] Department of Herpetology

Chengdu Institute of Biology

Chengdu, Sichuan, CHINA

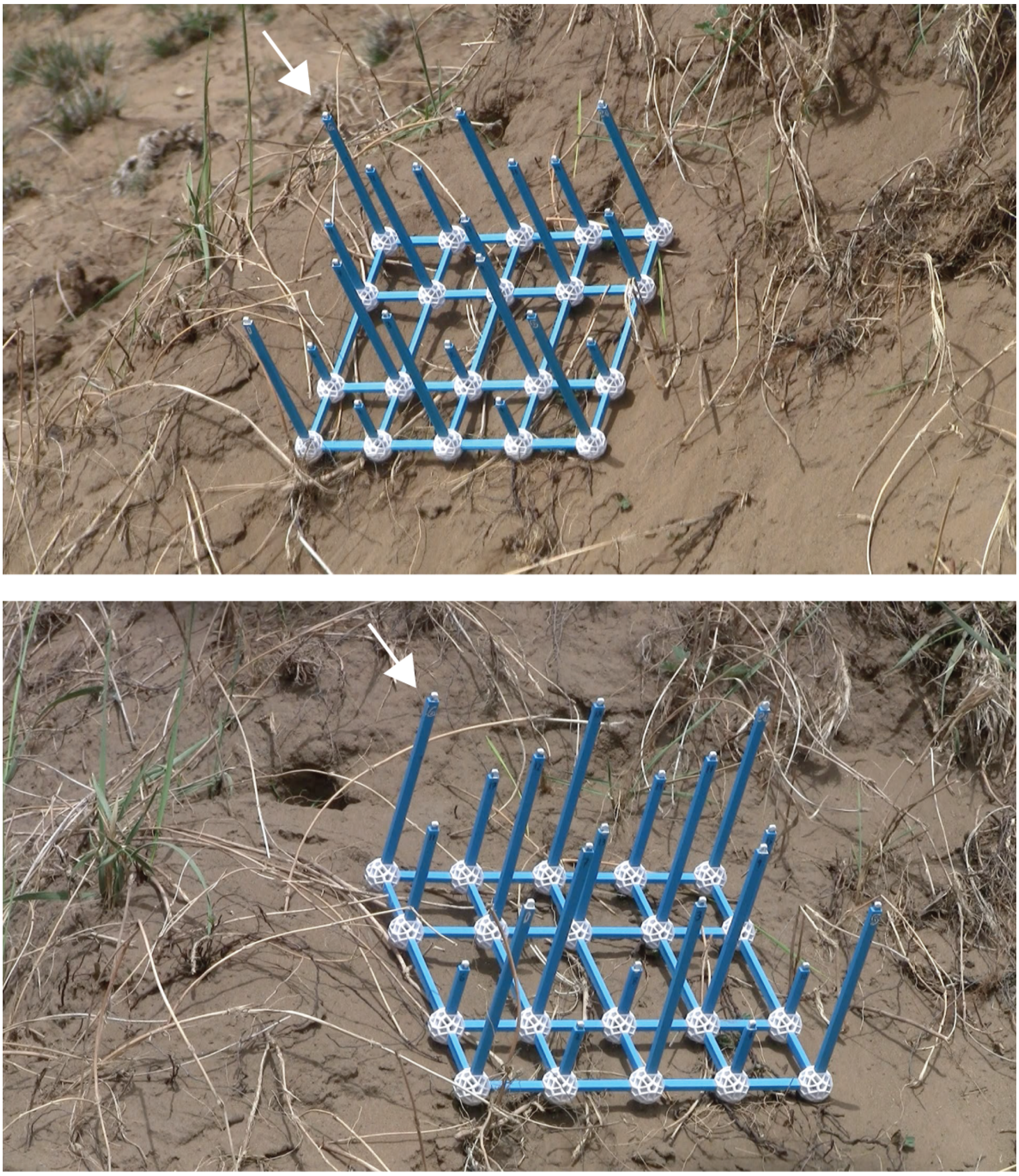

Figure S1

Photo of the calibration object used to facilitate three-dimensional reconstruction of tail movements. The object featured 20 points in a 5 x 4 grid at three different heights. Images are shown from two cameras placed at different viewing positions. Arrows indicate corresponding vertex in each image. The calibration object was created using ball and strut combinations of the Zometool construction system (Zometool Inc).

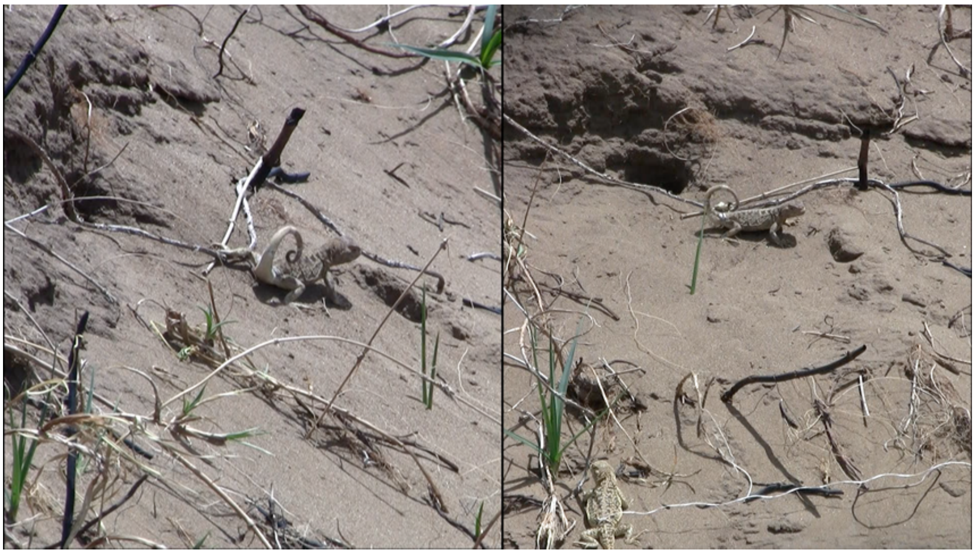

Movie S1

Video showing the three different display types used by *Phrynocephalus vlangalii* in this study. The first clip shows tail waving by a juvenile lizard, a putative submissive signal. This is followed by tail coiling and tail lashing by a female and male lizard respectively, which are used in burrow defense. Each tail display is presented as a split screen from two camera angles. (Image shows a single frame from Movie S1).
